# Sex Specificity in Innate Immunity of Insect Larvae

**DOI:** 10.1093/jisesa/ieab097

**Published:** 2021-12-04

**Authors:** Irina Belousova, Sergey Pavlushin, Anna Subbotina, Natalya Rudneva, Vyacheslav Martemyanov

**Affiliations:** 1 Institute of Systematics and Ecology of Animals SB RAS, Frunze str. 11, Novosibirsk 630091, Russia; 2 Biological Institute, National Research Tomsk State University, Lenin Ave. 36, Tomsk, 634050, Russia; 3 Reshetnev Siberian State University of Science and Technology, Krasnoyarsky Rabochy Ave. 31, Krasnoyarsk 660037, Russia

**Keywords:** sex, lysozyme, *Lymantria dispar*

## Abstract

The innate immunity of insects has been widely studied. Although the effect of sex on insect immunity has been extensively discussed, differences in immunity between the sexes of larvae insects remain largely unstudied. Studying larval sex differences in immunity may provide valuable information about the mechanisms underlying the insect immune system, which, in turn, can be valuable for the development and improvement of pest management. Here we compared the antibacterial activity in both the midgut tissue and cell-free hemolymph of *Lymantria dispar* L. (Lepidoptera: Erebidae) females and males at the larval stage without and after a challenge by entomopathogenic bacterium *Bacillus thuringiensis* Berliner. We also evaluated the sex-specific mortality of *L. dispar* induced by *B. thuringiensis* infection. We find that antibacterial activity in the midgut is activated by infection, but only in females. Thus, sex differences in immunity can have important effects even before sexual differentiation at adulthood.

There is a widespread view that males are the material for natural selection and ‘the sicker sex’ (Zuk, 2009). Females benefit from increased investment in longevity and therefore in immunity, whereas males invest their resources in reproduction (Zuk and McKean, 1996; Nunn et al., 2009). Despite widespread acceptance of this theory, the empirical data are ambiguous. Kelly et al. (2018) demonstrated the lack of a strong overall sex difference in the immunocompetence of animals in general. For insects, many studies illustrate the significant variation in immune function among the sexes (Rolff, 2001; Vainio et al., 2004; Schwarzenbach et al., 2005; Steiger et al., 2011; Sevgili 2019). However, most of these studies only examined adult insects. Here we seek to examine whether sexual dimorphism in insect immunity is extended to larval insects. This is important from a plant protection point of view, as larval insects are responsible for more plant harm than adults. Furthermore, most insecticides and biological controls are developed to target these harmful larvae.

The demand for novel strategies to control insect pests is pressing as the use of chemical insecticides is predicted to decline and insect resistance to biologicals is increasing. Most current methods of pest control are aimed at directly destroying individuals by applying biologicals (Lacey et al., 2015). Such an approach leads to the mortality of most pests, but not the entire population. Surviving individuals will recover the population size in several subsequent generations. The speed of this recovery depends on many factors, with the sex ratio being one of the most important. The fewer females there are in a population, the slower the population recovers (Schowalter, 2006). One excellent example of the effectiveness of biocontrol directed at female mortality is the ‘sterile insect technique’ (Marec et al., 2005). Theoretically, another new technique that could combine the rapid effect of biologicals and the targeting of females will significantly prolong the regulating effect of pest control and consequently slow the speed of insect resistance against biologicals. Given this view, understanding the immune function of larvae, and any immune differences between the sexes, is particularly important.

The questions of how widespread immune sexual dimorphism is in insects and whether it is possible to use this aspect for pest control are still open. To initiate the direction for new studies in this area, we carried out a study to estimate the sex-specific variation in an important parameters of innate immunity—antibacterial activity. We measured this parameter in the midgut tissue and cell-free hemolymph of the major lepidopteran pest species, *Lymantria dispar* L., larvae without and after challenge by *Bacillus thuringiensis*. We also evaluated the sex-specific mortality of *L. dispar* induced by *B. thuringiensis* infection.

## Materials and Methods

### Insects

The experiments were conducted at the Karasuk Research Station of the Institute of Systematics and Ecology of Animals, SB RAS, Western Siberia, Russia (53°42′N 77°45′E). *Lymantria dispar* eggs were collected in the spring from wild birch stands when the population was at the rising (prepeak) phase of the population cycle. Then, eggs were kept in a refrigerator at 4°C. The hatching of larvae was synchronized with the budburst of birch leaves (first third of May) in accordance with Martemyanov et al. (2015). After hatching, insects were reared on cut branches of silver birch *Betula pendula* Roth. Larvae were reared under laboratory conditions (21°С) in plastic containers, 50 larvae per 20-liter container at natural humidity and natural photoperiod.

### Challenge with *B. thuringiensis*

One day after molting, fourth-instar larvae were used for the bacterial infection. The peroral larval challenge was performed with *B. thuringiensis* ssp. *kurstaki* 2127 kindly provided by the museum of the Laboratory of Insect Pathology of the Institute of Systematics and Ecology of Animals, SB RAS. A water suspension of spores and crystals mixture (1:1) was evenly applied on the leaf surface by brush. The same number of leaves treated with water was used as the control. Leaves treated by bacteria or water were given to insects at the ratio of 20 leaves per 50 larvae. We used three concentrations for challenging: ‘low’ (3 × 10^7^ CFU/ml), ‘middle’ (5 × 10^7^ CFU/ml), and ‘high’ (10^8^ CFU/ml). Each leaf was treated with 0.33 ml of the bacterial suspension. A low concentration of bacteria was used to exclude the effect of selection on antibacterial activity, whereas middle and high concentrations were used to increase the resolution of sex-specific mortality. The sampling for the antibacterial study was 30 larvae/treatment, larvae fed on leaves from three replicates of trees. Sampling for the mortality study was as follows: 30 larvae/tree/treatment, 3 replicates of trees for the low concentration; 100 larvae/tree/treatment, 3 replicates of trees for middle concentration; and 40 larvae/tree/treatment, five replicates of trees for the high concentration. Mortality was recoded daily until adults emerged. The antibacterial activity was measured 1 d after the *B. thuringiensis* treatment/water (Martemyanov et al., 2016).

### Antibacterial Activity

The antibacterial activity was measured in the midgut tissue and cell-free hemolymph. The hemolymph of the larvae was collected with a micropipette through a puncture in the cuticle, placed in a cooled plastic centrifuge tube with a crystal of phenylthiourea, and centrifuged at 500 g for 10 min to free the plasma from the hemocytes. The midgut was dissected, and gut lumen contents covered with the peritrophic membrane were carefully removed. The tissue was homogenized by sonication in 50 μl of cold phosphate buffer with 0.15 М NaCl. Antibacterial activity was estimated against the bacterium *Micrococcus lysodeikticus* (Lee et al., 2006). Five microliters of cell-free hemolymph or supernatant of the midgut suspension was placed into holes in agar medium containing freeze-dried test microorganisms and incubated for 24 h at 28°С. Data were recorded as the areas of the lytic zones, which were photographed using a digital camera (Canon, Japan) and then measured with ImageJ software. Standard curves were obtained from a serial dilution of hen egg-white lysozyme equivalents (Sigma, USA). Antibacterial activity was normalized for 1 mg of protein. The protein concentration in the samples was quantified by the Bradford method.

### Sexing of Insects

An adult sex determination was performed using morphological features, and differences in the structures of the antenna (Pogue and Schaefer, 2007). Larvae sex determination was carried out using a qPCR technique (Belousova et al., 2019). Sex was determined by the ratio of the numbers of sex chromosomes (Z chromosome) to the autosome. DNA was extracted from the larval midgut by standard phenol–chloroform procedure. The qPCR mixture consisted of (2×) BioMaster HS-qPCR SYBR Blue (BioLabMix, Russia), 50 ng of DNA, and 100 nM (for *kettin*) or 50 nM (for *cad*) each primer. The reactions were run under the following conditions: 95°C for 5 min and 40 cycles of 95°C for 15 s and 58°C for 40 s. In the current study, we used modified specific primers for the gene of the sex chromosome *kettin* (TACAGC**T**AGCTC**T**CGAATC, GCCCGTAGGTGCATGATGTT, two mismatches on one of the original primers) to decrease the number of replicates. This was carried out to improve the accuracy according to recommendations in Belousova et al. (2019). Primers for the reference gene of the autosome (*cad*) were TGTTTCTACTCCTTCGTTTTAGGGG, and TGGGGTTAAATACTTTTGGTGCCT. Sex was determined by *∆Cq* (*Cq*_kettin_ − *Cq*_cad_).

### Statistical Analysis

The effects of sex and the *B. thuringiensis* infection on antibacterial activity were tested using the nonparametric Scheirer–Ray–Hare test (AtteStat 12.5), followed by Dunn’s post hoc test (PAST 3). Mortality and percent of surviving females were tested using a Mann–Whitney *U* test (Statistica 6.0). Data were checked for extremes.

## Results and Discussion

**Fig. 1.**
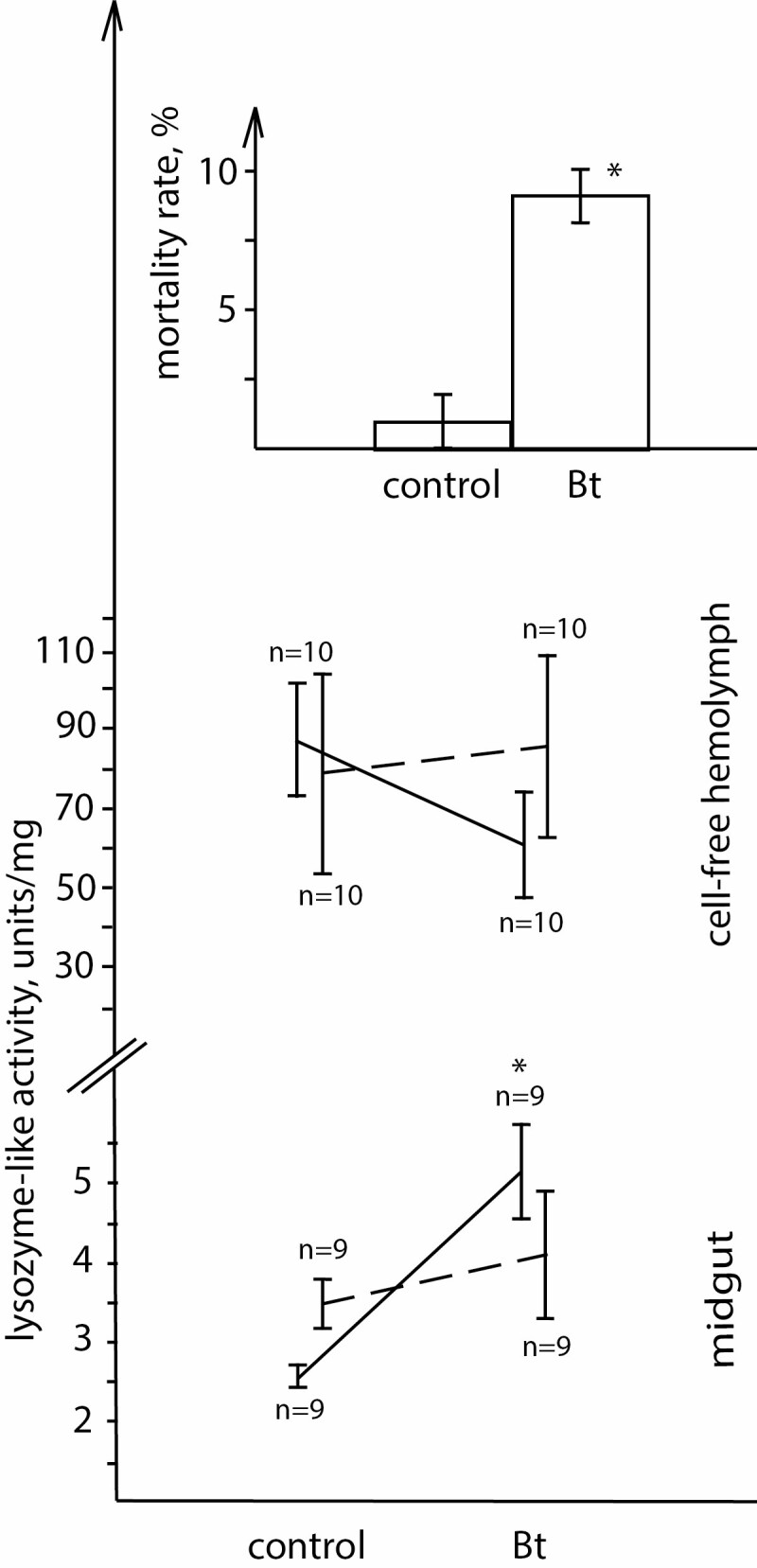
Percent mortality and antibacterial activity of cell-free hemolymph and midgut tissue in the control and after exposure to the low dose of *B. thuringiensis* (solid female, dashed male; *P < 0.05 in comparison with unexposed ‘control’ for mortality, *P < 0.05 in comparison with ‘female-control’ for antibacterial activity).

**Fig. 2.**
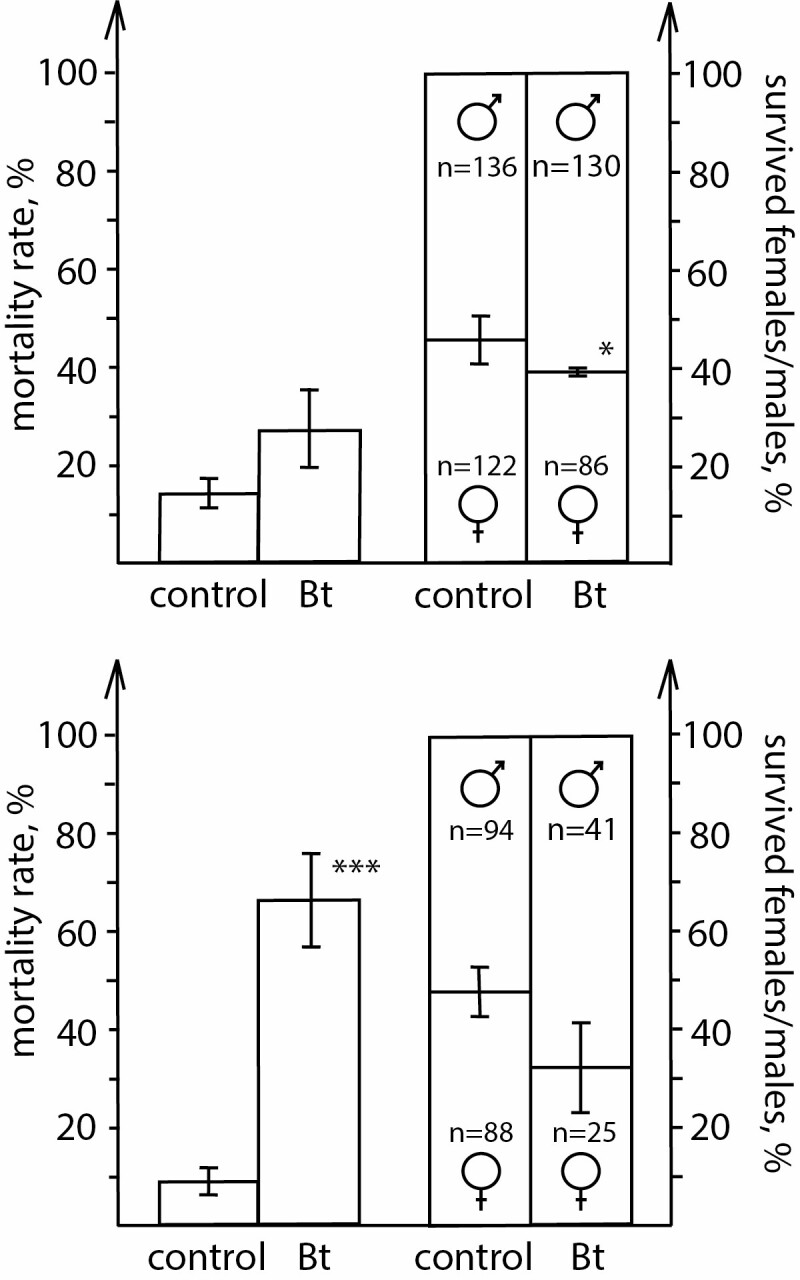
Percentage of surviving females/males relative to both sexes at middle dose (top) and high dose (bottom) of *B. thuringiensis* exposure (*P < 0.05, ***P < 0.001 in comparison with ‘control’).

Exposure to the low dose of *B. thuringiensis* resulted in 8.9% mortality (control-Bt: *Z* = −1.964, *P* = 0.049); 28% at the middle concentration (control-Bt: *Z* = −1.091, *P* = 0.27), and 67% at the high concentration (control-Bt: *Z* = −2.61116, *P* = 0.009; Figs. 1 and 2).

Antibacterial activity significantly increased in the midgut tissue of the infected larvae (low concentration of bacteria, without accounting for sex) relative to uninfected control individuals (*H*_1,35_ = 3.973, *P* = 0.0462). Sexing of the tested samples showed that this increase of antibacterial activity was driven by females (female: control-Bt *P* = 0.0029; Sex × Bt *H*_1,35_ = 4.9049, *P* = 0.0268; Fig. 1). The activity of this enzyme in the midgut tissue of male larvae was not affected by infection (male: control-Bt *P* = 0.8756). An induction of antibacterial activity by *B. thuringiensis* challenge for unsexed insects has been described for many species, including *L. dispar* (Wang et al., 2010; Crava et al., 2015; Haloi et al., 2016; Martemyanov et al., 2016; Batool et al., 2018). However, our results demonstrate for the first time that only one sex (females in our study) may invest in this activation. This result means that when studying innate immunity, it is necessary to account for sex before drawing a conclusion about the effect of a pathogen on an insect population as a whole.

The antibacterial activity in the cell-free hemolymph of the larvae was not affected by bacterial infection or sex (Sex × Bt *H*_1,39_ = 1.8292, *P* = 0.1762; Fig. 1). The absence of a bacterial effect on this immune parameter contradicts data from Martemyanov et al. (2016) that could be related to the lower concentration of bacteria used for the current study. Kurtz et al. (2000) showed that the hemolymph of *Panorpa vulgaris* derived from larval females had higher antibacterial activity than that derived from males. Our data together with the work of Kurtz and coauthors indicate that antibacterial activity may be considered as a sex-specific immune parameter at the larval stage.

Among larvae given the middle concentration of bacteria, a lower percent of females survived than males (*Z* = 1.963, *P* = 0.049; Fig. 2), but this difference was no longer significant at high dose (*Z* = 1.253, *P* = 0.21; Fig. 2), although the tendency was the same. Other studies describe minimal differences between males and females in infection susceptibility. For example, in a meta-analysis, Sheridan et al. (2000) found no differences in the prevalence or intensity of parasite infections between the sexes of arthropods. However, a number of individual studies have found an infection bias toward one sex or the other including studies on insect larvae (Gray, 1998; Morton and Garcia-del-Pino, 2013). Correspondingly, Sheridan et al.’s conclusion of no sex specificity may be premature.

The literature has suggested a difference in how males and females invest in life-history traits. Traditionally, this is viewed as males investing more in reproduction, and less in maintenance tasks such as maintaining or mounting a strong immune response. Our data on immunity support the theory that males choose investment in reproduction over investment in immunity. However, *B. thuringiensis*-induced mortality data show the opposite effect that while females mount a stronger immune response, they also suffer higher mortality. This apparent incongruity may reflect the limited resources in general and their redistribution directly to the reproductive system. It is worth noting that the bacterial induced mortality in females could be even higher if they did not invest in increased in immune responses to bacterial exposure.

For pest control, the concept of manipulating insect innate immunity has been widespread among researchers for many years. For example, selected brighteners significantly enhanced the virulence of the gypsy moth nucleopolyhedrovirus owing to effects on apoptosis (Dougherty et al., 2006). Azadirachtin possesses immunosuppressive effects and provides synergism when it is used together with *B. thuringiensis* (Nouri-Ganbalani et al., 2016; Dorrah et al., 2019). Immune-related genes have been recently identified as potential RNAi targets for controlling insects (Ran et al., 2018). Thus, if researchers combine the concept of manipulating innate immunity with the concept of prolonging the effect of biologicals (owing to a decrease of female proportions), the benefit could be particularly high. This effect can be achieved by increasing mortality in females by suppressing the immune parameters that have a higher activity in females than in males. According to the results of this study, the target immune parameter for this approach may be the antibacterial activity of the midgut.

### Conclusions

We showed that an increase of the antibacterial activity in the midgut of *L. dispar* larvae activated by a *B. thuringiensis* infection occurs only in females. We state that this parameter of immunity can be sex specific, moreover, occur at the juvenile stage. Additionally, sex-specific differences have been identified for *B. thuringiensis*-induced mortality among *L. dispar* larvae.
